# Perceived personal deadlines for late-life preparation across adulthood

**DOI:** 10.1007/s10433-020-00581-8

**Published:** 2020-09-28

**Authors:** Frieder R. Lang, Fiona S. Rupprecht

**Affiliations:** Institute of Psychogerontology, Friedrich-Alexander University of Erlangen-Nuremberg (FAU), Kobergerstr. 62, 90408 Nuremberg, Germany

**Keywords:** Perceived deadlines, Preparation for old age, End of life, Domain specificity, Subjective position in life

## Abstract

Throughout adulthood, individuals follow personal timetables of deadlines that shape the course of aging. We examine 6-year-longitudinal data of perceived personal deadlines for starting with late-life preparation across adulthood. Findings are based on a sample of 518 adults between 18 and 88 years of age. Multilevel regression analyses were conducted to explore changes in personal deadlines for preparation in five domains (i.e., finances, end of life, housing, social connectedness, caregiving) in relation to calendar age, self-rated health, subjective position in life, and sociodemographic variables. Findings suggest that personal deadlines for starting preparatory activities differ depending on calendar age and domain of late-life preparation. Older adults as compared to younger adults are likely to report narrower deadlines for beginning with late-life preparation. Perceived deadlines for late-life preparation were furthermore found to be preponed and slightly dilated over time. Findings suggest that depending on age-graded opportunity structures, individuals flexibly adjust their personal deadlines for late-life preparation.

**Electronic supplementary material:**

The online version of this article (10.1007/s10433-020-00581-8) contains supplementary material, which is available to authorized users.

## Introduction

Preparation for late life has become a newly emerging developmental task in early, middle, and later adulthood in many modern societies (Adams and Rau 2011). Throughout adulthood, individuals may follow personal timetables of perceived deadlines that shape the course of aging (Settersten 2003). For example, individuals need to plan and decide about when to engage in preparatory activities and may thus create perceived timetables of deadlines for late-life preparation. The timing of preparatory activities should hereby depend on the task at hand and the respective life domain. Some preparatory activities may require engagement over extended periods and should therefore be started early in life, and at times, one may also think that it is too late to still begin with a preparatory investment in a certain domain. For example, financial planning for later adulthood may require that individuals start planning early in life, and may also involve perceptions that there comes a time in life, when it appears impossible to catch up on delays in preparatory financial investment. In accordance with the idea that individuals are co-constructing their own development across adulthood and old age (Lang et al. 2011; Lerner and Busch-Rossnagel 1981), it has been shown that attitudes toward preparing for aging and old age depend on the specific domain of preparation (Kornadt and Rothermund 2014) such as finances (e.g., having a private old age pension; Street and Desai 2011), need of care (e.g., deciding on care preferences; Pinquart and Sörensen 2002), living arrangements (e.g., planning for age-appropriate housing; Gibler and Lee 2005), social connectedness (e.g., investing in social relationships; Nimrod et al. 2009), and end-of-life decisions (e.g., writing a last will; Steinhauser et al. 2001).

Late-life preparation was defined as “an effort to ensure that major problems will not develop at some point in the distant future” (Jacobs-Lawson et al. 2004, p. 57). Consequently, seeking to minimize potential future threats while maximizing positive outcomes also involves time-related perceptions about when it is adequate to engage in such preparatory activity. In particular, we explore perceived deadlines for late-life preparation across adulthood. Not much is known about individuals’ perceptions of when is an adequate time in their life course to begin with preparation for late life. For example, when would be an adequate time in one’s life course to begin with financial planning or when should one begin preparing oneself for future needs of caregiving? Do deadlines for preparation across various domains differ from another? When is it adequate to start to plan for one’s end of life? Some may say that such issues become relevant only when approaching one’s end of life, while others may believe that one should start to prepare in midlife. Similarly, some may think that preparation for a possible future need of care, or for prevention of social isolation in late life should be started early in life. In our research, we explore associations of calendar age with perceived deadlines of preparation in five different domains of preparatory activities above and beyond other covariates such as self-rated health and subjective position in life (e.g., subjective age, perceived life expectancy). Furthermore, we explore the stability and change of such perceived personal deadlines at four measurement occasions across a 6 year time interval.

Models of developmental regulation in adulthood (Brandtstädter and Rothermund 2003; Heckhausen 2000) emphasize the role of developmental timing in the process of life-task accomplishments. Action theoretical models suggest that the implementation and realization of personal goals follow a personal timetable on how and when to engage in the planning and implementation of one’s future endeavors (Aspinwall and Taylor 1997; Löckenhoff et al. 2012). Accordingly, we propose that individuals may follow individual patterns of timing to decide about when and how to begin with preparations for their future late life. We focus on the time windows of when to start one’s preparations as defined by two points, that is, (a) the perceived earliest adequate point to begin, and (b) the perceived latest adequate point to begin with such preparatory activities for late life. This latter time point reflects a perceived deadline after that it may be too late to begin with preparations. We submit that individuals may adjust their personal deadlines over time depending on one’s calendar age and one’s current life situation. Not much is known about stability and change of such perceived deadlines across adulthood.

Building on assumptions of socioemotional selectivity theory (SST; Carstensen et al. 1999), we expect that changes in perceived personal deadlines for certain domains of late-life preparation reflect shifts in personal preferences and decisions across adulthood. For older adults, the domains of late-life preparation, which are associated with meaningful social contact and with the final phase of life (e.g., caregiving, social connectedness, end of life), may appear more salient and urgent relative to other domains of preparation (e.g., finances) that may be associated with long-term planning, and may thus be more salient for younger adults. In contrast, in early adulthood it may seem less relevant to conceive one’s last will or to arrange for caring needs.

We begin our research with the examination of change of domain-specific perceived personal deadlines of late-life preparation depending on type of domain (e.g., financial or end of life), calendar age, self-rated health, and subjective position in life (i.e., subjective age, perceived life expectancy, future time perspective). Moreover, possible changes in personal deadlines may reflect in what ways individuals adjust their personal deadlines of preparation to age-associated change across adulthood (Heckhausen 2000). For example, as time passes individuals may come to believe that starting earlier in life with preparation activities is more adequate. Younger adults may have a feeling that there remains more time in their life to prepare for old age and thus, have more liberal deadlines. In contrast, older adults may generally prefer more restrictive deadlines.

Furthermore, we also submit that the possible differences of deadlines between various domains of late-life preparation may also be associated with structures of social welfare. In Germany, social security and care insurance provides a social safety net with regard to the financial situation and provision of care in late life. In contrast, preparing for social connectedness, housing, and for end of life is mostly depending on personal efforts and investments in such domains. In the absence of prior research, we have no specific hypotheses with regard to domain-specific differences in perceived personal deadlines, but we expect that some domains may show greater malleability across adulthood and across time. For example, preparing for end-of-life issues may be adjusted more strongly over time than financial preparation for old age, which may be associated with earlier deadlines throughout adulthood.

### The present study

We are not aware of any prior research that has systematically examined perceived personal deadlines of late-life preparation across adulthood, across time, and across different domains of late-life preparation. In our research, we focus on age-associated shifts and change in perceived personal deadlines of when to begin with aging preparation in five domains. For this, participants reported perceived deadlines for beginning with late-life preparation activities on graphic life course scales ranging from birth to death. Consistent with the Theory of Socioemotional Selectivity (Carstensen et al. 1999; Lang and Carstensen 2002), we submit that perceptions of adequate timing of late-life preparation follow a more restrictive pattern of narrow deadlines in old age as compared to earlier adulthood. Approaching late life involves an understanding that preparing for this phase of life is becoming more relevant to the self over time. Furthermore, in old age individuals may be more likely to engage in those preparation activities that secure meaningful experiences. We explicitly decided to focus on perceived deadlines for the adequate (earliest and latest) beginning of preparation. By doing so, we submit that late life preparation reflects a lifetime task, whose beginning is mostly under an individual’s personal control. In order to explore the generality of the phenomenon that we observed, we also tested for possible effects of covariates such as subjective age, self-rated health, perceived life expectancy, and future time perspective, in addition to marital status, parental status, and retirement status. We did not assess perceptions of when preparatory activities should be completed as this depends on conditions that may not always be foreseeable (e.g., need of care, disease).

## Methods

### Sample and procedure

Data were collected via four longitudinal online studies taking place in the years 2012, 2014, 2016, and 2018 in Germany. At all four measurement occasions, new participants were recruited and longitudinal participants were invited to take part again. For our longitudinal analyses we only used data from those participants who took part in the study at least twice. This led to a sample size of 518 adults. Out of these, 150 (29%) participated in all four measurement occasions, 130 (25%) in three measurement occasions, and 238 (46%) in two measurement occasions. At their respective first participation (i.e., 2012, 2014, or 2016), participants were aged 18–88 years (*M* = 48.1; SD = 18.5). Among the participants, 64.5% were women, 31.7% were retired throughout their participation, and 31.5% were married throughout their participation.

### Variables

**Fig. 1:**
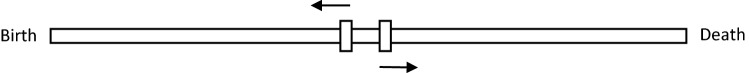
Graphic life course scale and item wordings. When is, in your opinion, the earliest (good) and the latest (still good) point in time that one should start preparing: (1) …for financial security in old age/(2) …for living arrangements in old age/(3) …for the possibility of needing caregiving/(4) …against loneliness in old age/(5) …for dying and death

As outcome variables we chose the earliest and the latest adequate time points (i.e., starting and ending points) to begin with aging preparation in the five domains finances, housing, care, social connectedness, and end of life. For example, we asked: “When is, in your opinion, the earliest (good) and the latest (still good) point in time that one should start preparing for dying and death?”. The participants indicated their answers to the respective item with two sliders on a graphic life course scale ranging from “birth” (0) to “death” (100), see Fig. 1. The questionnaire was structured into five domain-specific blocks of items ordered in the same way across measurement points. The question for the starting and ending points was the opening question of the respective item block and was presented after the domain was named. No examples on possible specific preparation activities within the respective domain were given.^1^

In order to validate the graphic life course scale, we first asked participants to indicate their own current life position by using one slider (cf. Cottle 1976). Indeed, the current life positions on the graphic scale and the calendar ages of the participants were strongly comparable, *r* = .90, *p* < .001. The midpoint of the graphic scale (50) did for example match a calendar age of 44.63 years. Over the study period of 6 years, individuals indicated increasingly later life positions on the graphic life course scale (*M* = +7.7 points, SD = 8.4 points). The scale hence appeared sensitive to the aging process and changes over time. Consequently, the graphic life course scale may allow to assess issues of timing without explicitly asking for age deadlines in number of years.

All available starting and ending points from the measurement occasions 0, 1, 2, and 3 were used in order to investigate time trends. With regard to predictor variables and covariates, we only used the earliest available values of an individual, that is, the values from the first measurement occasion at which the respective individual had joined the study. We did so as we were primarily interested in how earlier values shape the starting and ending points over time. As the main predictor variable we chose calendar age, indicated as years lived since birth. We investigated how calendar age was related to starting and ending points for late-life preparation overall, over time (i.e., in interaction with the time variable), as well as specifically for certain domains.

In an attempt to rule out that the relationship between objective aspects of life positioning (i.e., calendar age) on perceived personal deadlines is confounding with effects of subjective positioning within the life course, we added subjective age, self-rated health, perceived life expectation, and future time perspective to our calculations as covariates. Subjective age was calculated as the discrepancy between the age an individual feels like and the respective individual’s calendar age divided by calendar age (cf. Rubin and Berntsen 2006). A subjective age score of − .10 would hence indicate that an individual feels 10% younger than his or her calendar age. Subjective age was truncated at + .50 (*N* = 7). Hence, lower subjective age scores hint to an earlier subjective positioning within the own life course. Self-rated health ranged from 1 (bad) to 5 (very good). Perceived life expectancy was assessed with the question “To what age do you expect to live?” (Lang and Rupprecht 2019). Future time perspective (FTP) was assessed with the 10-item future time perspective scale by Carstensen and Lang (1996). Individuals indicated their agreement with statements such as “My future seems infinite to me” on a scale ranging from “does not apply at all” (1) to “applies very much” (7). Cronbach’s alpha was .89 and a lower future time perspective hints to a later subjective positioning within the life course. Bivariate correlations among the domain-specific starting and ending points and these non-demographic covariates can be found in Supplementary Table S1.

Lastly, we entered further covariates to the regression models, that is, sex, marital status, parental status, retirement status, and household net income. Sex was coded as 0 for men and 1 for women. In regard to marital status, parental status, and retirement status, 0 indicated that participants were unmarried, had no children, and were not retired, whereas 1 indicated the respective opposites. Household net income ranged from 1 (less than 1000€) to 7 (6000€ or more). Missing values in household income (*N* = 9) were estimated based on variables such as saving behavior and the possession of real estate.

### Data analysis

Data analyses were conducted with two three-level regression models, one with starting points for late-life preparation as the outcome variable, and one with ending points for late-life preparation as the outcome variable. Level 3 differentiates between individuals. Level 2 differentiates the four measurement occasions (0, 1, 2, or 3) using a continuous time variable. Level 1 differentiates the five domains of preparation, which were assessed for each individual at each participation. Finances served as the reference category and the four other domains were indicated as dichotomous dummy variables.

We investigated overall linear time trends in starting and ending points using the time variable (0, 1, 2, 3). The covariates and calendar age were entered as predictors on the highest level (i.e., the individual level). To test whether starting and ending points in some domains developed differently over time than in others, we included a time * domain interaction to the regression models. To test whether calendar age influenced starting and ending points differently for the five domains as well as over time, we investigated the interactions age * domain, age * time, and age * domain * time. Except for time and the domain dummy variables, all predictor variables were grand-mean centered before we entered them into the multilevel regression model.

Finances served as the reference category; therefore, the intercept reflects the starting or ending point estimated for an individual with average values on all included predictor variables and covariates at measurement occasion 0 in the domain of finances. We labeled this intercept as *γ* = *γ*_Finances_. The main effects of the domains (Models 1–5) were labeled as deltas (*δ*_Housing_, *δ*_Care_, *δ*_Loneliness_, and *δ*_EndofLife_), as they describe deviations from *γ* instead of the total effects of the domains. These total effects of the domains can for example be calculated by *γ*_Dying_ = *γ *+ *δ*_EndofLife_.^2^

Five successive models were estimated for both starting and ending points with an increasing number of predictor variables, covariates, and interactions. For estimating the models we used R 3.4.3 (R Core Team 2020) and the R packages MuMIn, lme4, reghelper, and lmerTest (Barton 2020; Bates et al. 2015; Hughes 2020; Kuznetsova et al. 2017). The models were estimated using restricted maximum likelihood (REML) and *p* values were calculated with the Satterthwaite method. For model comparison, the Akaike information criterion (AIC), the Bayesian information criterion (BIC), and marginal *R*^2^ are provided.

## Results

### Predicting starting points for aging preparation

The intercept in Table 1, Model 1, gives the average starting point in the domain of financial preparation at measurement occasion 0 and is estimated as *γ* = *γ*_Finances_ = 29.76 (SE = 0.68, *p* < .001) on the graphic life course scale ranging from 0 to 100. The starting points in the other four domains were all set significantly later. In the domains housing, care, and end of life, the starting points were hereby all set around the midpoint of the graphic scale, e.g., *γ*_EndofLife_ = *γ* + *δ*_EndofLife_ = 29.76 + 18.66 = 48.32. Over time and averaged across domains, starting points were set increasingly earlier, i.e., *γ*_Time_ = −2.71 points per measurement occasion and hence, every 2 years. On the individual level, starting points were weakly to moderately stable over the 6 years of the study: The 6-year rank-order stability of the starting points was .48 across domains, and .43, .14, .34, .40, and .34 within the domains of finances, housing, care, connectedness, and end of life, all *p* values < .05.^3^

**Table 1:** Predicting starting points of aging preparation across domains, over time, and nested in individuals

	Model 1	Model 2	Model 3	Model 4	Model 5
Intercept	29.76* (0.68)	29.43* (0.68)	29.54* (0.68)	25.51* (0.90)	25.73* (0.92)
Time	− 2.71* (0.21)	− 2.55* (0.22)	− 2.55* (0.22)	− 0.15 (0.42)	− 0.22 (0.42)
Housing	21.36* (0.62)	21.37* (0.62)	21.39* (0.62)	32.33* (1.11)	32.72* (1.14)
Care	19.04* (0.62)	19.06* (0.62)	18.92* (0.62)	22.85* (1.11)	22.74* (1.14)
Connectedness^a^	8.46* (0.62)	8.45* (0.62)	8.24* (0.62)	11.82* (1.11)	11.71* (1.14)
End of life^b^	18.66* (0.62)	18.62* (0.62)	18.40* (0.62)	19.19* (1.11)	17.95* (1.14)
Age		0.10* (0.05)	0.02 (0.05)	0.08 (0.06)	0.03 (0.07)
FTP		− 1.27* (0.45)	− 1.27* (0.45)	− 1.27* (0.45)	− 1.27* (0.45)
Age * housing			− 0.01 (0.03)	− 0.09* (0.03)	− 0.17* (0.06)
Age * care			0.10* (0.03)	0.07* (0.03)	0.09 (0.06)
Age * connected^a^			0.15* (0.03)	0.12* (0.03)	0.15* (0.06)
Age * end of life^b^			0.15* (0.03)	0.15* (0.03)	0.40* (0.06)
Time * age				− 0.02 (0.01)	0.01 (0.02)
Time * housing				− 6.67* (0.57)	− 6.80* (0.57)
Time * care				− 2.39* (0.57)	− 2.36* (0.57)
Time * connected^a^				− 2.19* (0.57)	− 2.15* (0.57)
Time * end of life^b^				− 0.48 (0.57)	− 0.07 (0.57)
Time * age * H					0.05 (0.03)
Time * age * C					− 0.01 (0.03)
Time * age * SC^a^					− 0.01 (0.03)
Time * age * EoL^b^					− 0.15* (0.03)
*σ*_Intercept_^2^	70.11	63.76	63.79	63.43	63.42
*σ*_Time_^2^	12.34	12.55	12.91	14.57	14.93
*σ*_Domain_^2^	282.85	282.85	280.98	273.08	271.33
Marginal *R*^2^	16.92%	18.84%	19.20%	20.68%	21.03%
AIC	62,910.81	62,800.68	62,787.03	62,630.31	62,618.47
BIC	62,972.85	62,931.57	62,945.45	62,823.12	62,838.80

Standard errors are in parentheses. *FTP* future time perspective, *H* housing, *C* care, *SC* social connectedness, *EoL* end of life, *AIC* Akaike information criterion, *BIC* Bayesian information criterion. Finances serve as the reference category. In Model 2, Model 3, Model 4, and Model 5, sex, marital status, parental status, retirement status, household net income, self-rated health, perceived life expectancy, and subjective age are added as covariates but do not reach significance. The inclusion and exclusion of those covariates did not change the effects of the main predictors and interactions

*p < .05

^a^The domain of social connectedness was assessed as preparation *against* loneliness in old age

^b^The end-of-life domain refers to preparation for dying and death

**Fig. 2:**
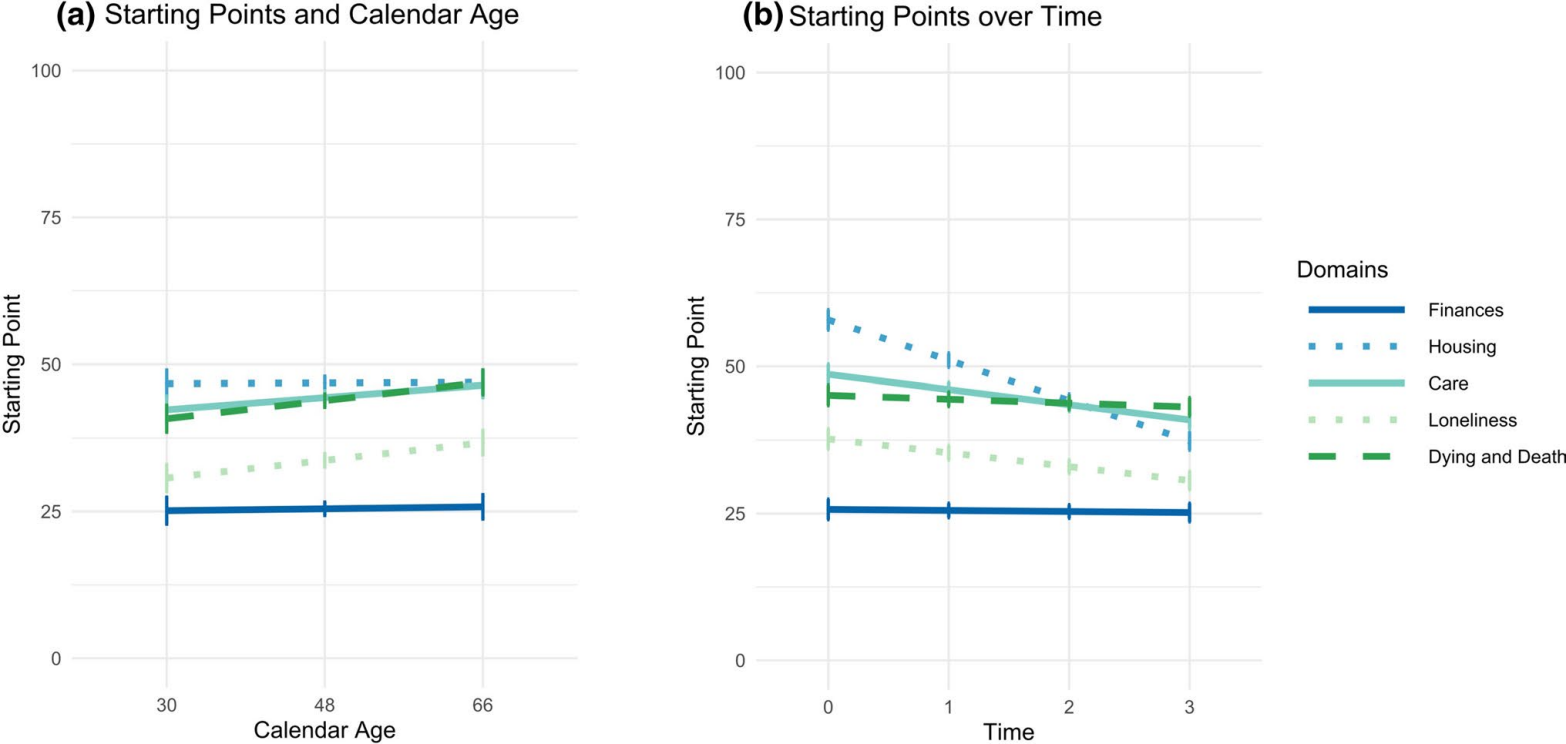
Domain-specific starting points and their relations to time and age. *Notes* Starting points are depicted for the five domains finances, housing, care, connectedness, and end of life and can range from 0 (birth) to 100 (death). **a** The domain-specific effects of time on the starting points (Table 1, Model 3), **b** the domain-specific effects relations between calendar age and the starting points (Table 1, Model 4)

Calendar age was significantly associated with the starting points across domains (Table 1, Model 2). Namely, older participants presented overall later starting points for aging preparation, *γ*_Age_ = 0.10, SE = 0.05, *p* = .049. When relations between calendar age and starting points were investigated specifically for the five domains (Table 1, Model 3), the relation was nonsignificant in the reference domain of finances, *γ*_Age_ = *γ*_Age:Finances_ = 0.02 (SE = 0.05, *p* = .732). The relation to calendar age was comparable and also nonsignificant for the domain of housing, but stronger and therefore significant in the domains of care, social connectedness (i.e., not being lonely), and end of life (Fig. 2a). Hence, older participants chose later starting points for preparing for care, social connectedness, and end of life than younger participants. Additionally, above and beyond such effects, future time perspective reached significance as a covariate, *γ*_FTP_ = −1.27, SE = 0.45, *p* = .005. A more narrow future time perspective was hence associated with later starting points. None of the other covariates, all *p* values > .080, was significantly related to the starting points.

The time trends in the starting points differed by domain (Table 1, Model 4). There was no general linear time trend in the domain of finances, *γ*_Time_ = *γ*_Time:Finances_ = −0.15, SE = 0.42, *p* = .715, and there was also none in the end-of-life domain. The linear time trends were however stronger and therefore significant in the domains housing, care, and social connectedness (see Fig. 2b). Over time, starting points were set increasingly earlier in those domains. The most pronounced change thereby appeared in the domain of housing, in which starting points decreased by *γ*_Time:Housing_ = *γ*_Time_ + *δ*_Time:Housing_ = −0.15 + (−6.67) = −6.82 per year, and hence by − 20.46 over the 6 years of the study. The starting points for housing were thus located in the second half of the graphic scale in 2012 and 2014, but in the first half of the graphic scale in 2016 and 2018.

**Fig. 3:**
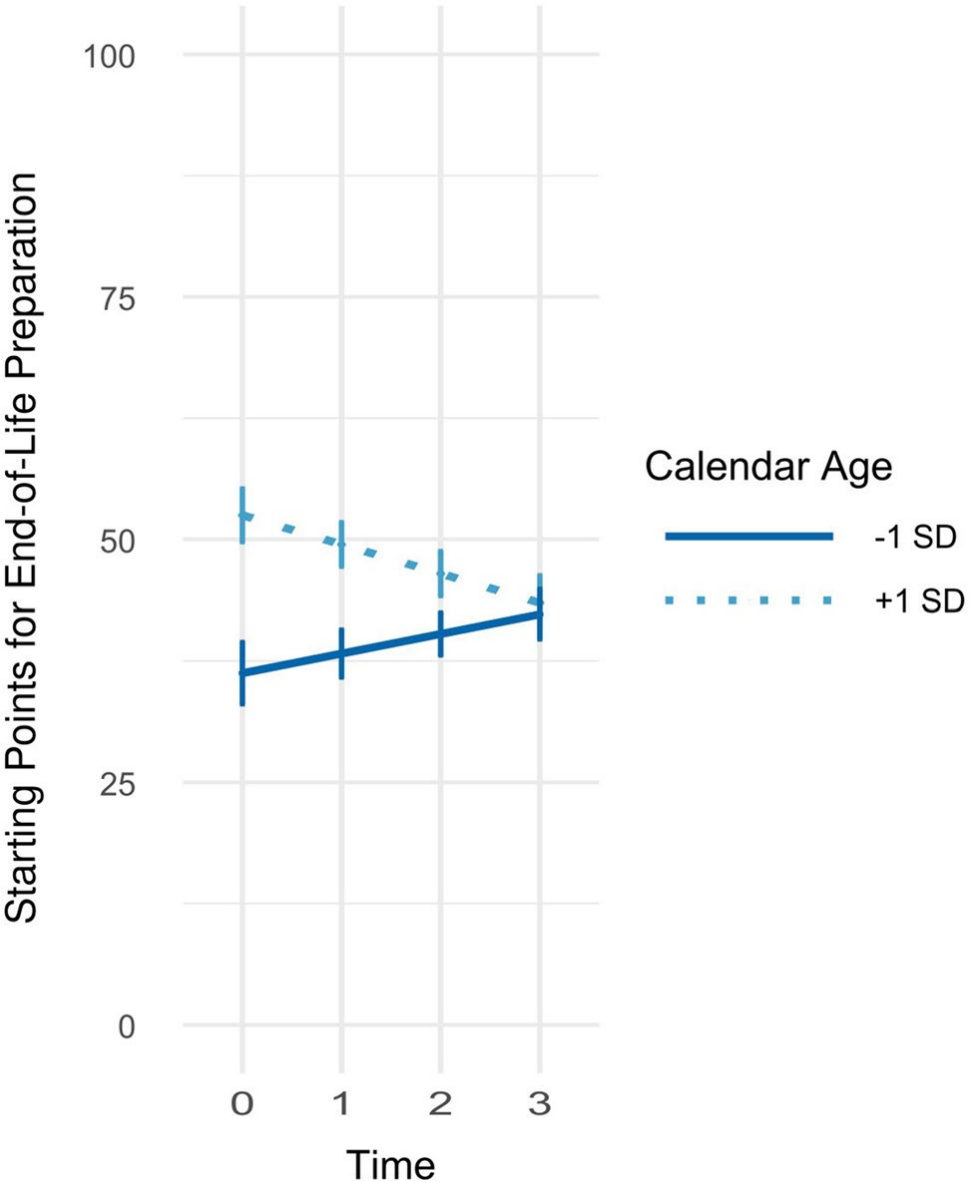
Age-specific time trends in starting points in preparation for end of life. *Notes* Starting points can range from 0 (birth) to 100 (death). The figure shows how time trends in the starting points for dying and death preparation are influenced by calendar age (Table 1, Model 5)

Calendar age did not affect general time trends in the starting points, *γ*_Time * Age_ = −0.02, SE = 0.01, *p* = .185, (Table 1, Model 4). When investigated specifically for the five domains, only in the domain of end-of-life preparation the linear time trend interacted with age, *δ*_Time * Age:EndofLife_ = −0.15, SE = 0.03, *p* < .001 (Table 1, Model 5). Precisely, younger participants set increasingly later starting points over time, whereas older participants set increasingly earlier starting points for end-of-life preparation over time (see Fig. 3).

Taken together, the starting points for aging preparation were set differently depending on the domain and were set the earliest for the domain of finances. Over the course of the study, starting points for aging preparation became earlier overall, but especially in the domain of housing. An older calendar age and a more narrow future time perspective were both related to later starting points. They did not affect general time trends. However, age seemed to affect time trends specifically in the end-of-life domain. Altogether, 21.0% of variance in the starting points could be explained.

### Predicting ending points for aging preparation

The intercept in Table 2, Model 1, gives the average ending point in the domain of financial preparation at measurement occasion 0 and is estimated as *γ* = *γ*_Finances_ = 59.09 (SE = 0.63, *p* < .001) on the graphic life course scale ranging from 0 to 100. The ending points in the four other domains were all set significantly later and within the last quarter of the graphic scale, e.g., *γ*_EndofLife_ = *γ* + *δ*_EndofLife_ = 59.09 + 26.98 = 86.07. Over time and averaged across domains, ending points were set slightly but significantly earlier by *γ*_Time_ = −0.60 points per measurement occasion and hence, every 2 years. On the individual level, ending points were moderately stable over the 6 years of the study: The 6-year rank-order stability of the ending points was .52 across domains, and .35, .24, .33, .45, and .44 within the domains of finances, housing, care, connectedness, and end of life, all *p* values < .001.^4^

**Table 2:** Predicting ending points of aging preparation across domains, over time, and nested in individuals

	Model 1	Model 2	Model 3	Model 4	Model 5
Intercept	59.09* (0.63)	59.55* (0.62)	59.34* (0.62)	56.82* (0.76)	56.91* (0.77)
Time	− 0.60* (0.23)	− 0.83* (0.23)	− 0.83* (0.23)	0.72* (0.35)	0.68 (0.35)
Housing	17.05* (0.45)	17.04* (0.45)	17.18* (0.45)	23.08* (0.81)	23.13* (0.83)
Care	17.06* (0.45)	17.07* (0.45)	17.19* (0.45)	19.55* (0.81)	19.58* (0.83)
Connectedness^a^	21.34* (0.45)	21.34* (0.45)	21.79* (0.45)	22.93* (0.81)	22.83* (0.83)
End of life^b^	26.98* (0.45)	26.96* (0.45)	27.35* (0.45)	30.61* (0.81)	30.22* (0.83)
Age		− 0.15* (0.05)	0.00 (0.05)	0.02 (0.05)	− 0.00 (0.06)
Age * housing			− 0.10* (0.02)	− 0.14* (0.02)	− 0.15* (0.05)
Age * care			− 0.09* (0.02)	− 0.10* (0.02)	− 0.11* (0.05)
Age * connected^a^			− 0.31* (0.02)	− 0.32* (0.02)	− 0.30* (0.05)
Age * end of life^b^			− 0.27* (0.02)	− 0.29* (0.02)	− 0.21* (0.05)
Time * age				0.00 (0.01)	0.01 (0.02)
Time * housing				− 3.60* (0.41)	− 3.61* (0.41)
Time * care				− 1.44* (0.41)	− 1.45* (0.41)
Time * connected^a^				− 0.69 (0.41)	− 0.66 (0.41)
Time * end of life^b^				− 1.99* (0.41)	− 1.86* (0.41)
Time * age * H					0.01 (0.02)
Time * age * C					0.00 (0.02)
Time * age * SC^a^					− 0.01 (0.02)
Time * age * EoL^b^					− 0.05* (0.02)
*σ*_Intercept_^2^	60.48	49.60	49.56	49.54	49.54
*σ*_Time_^2^	49.78	49.48	50.66	51.14	51.15
*σ*_Domain_^2^	150.60	150.54	144.78	142.68	142.60
Marginal *R*^2^	23.87%	27.34%	28.71%	29.21%	29.24%
AIC	59,342.62	59,197.46	58,997.63	58,926.20	58,951.10
BIC	59,404.65	59,328.35	59,156.05	59,119.02	59,171.42

Standard errors are in parentheses. *H* housing, *C* care, *SC* social connectedness, *EoL* end of life, *AIC* Akaike information criterion, *BIC* Bayesian information criterion. Finances serve as the reference category. In Model 2, Model 3, Model 4, and Model 5, sex, marital status, parental status, retirement status, household net income, self-rated health, perceived life expectancy, subjective age, and future time perspective are added as covariates but do not reach significance. The inclusion and exclusion of those covariates did not change the effects of the main predictors and interactions

*p < .05

^a^The domain of social connectedness was assessed as preparation *against* loneliness in old age

^b^The end-of-life domain refers to preparation for dying & death

**Fig. 4:**
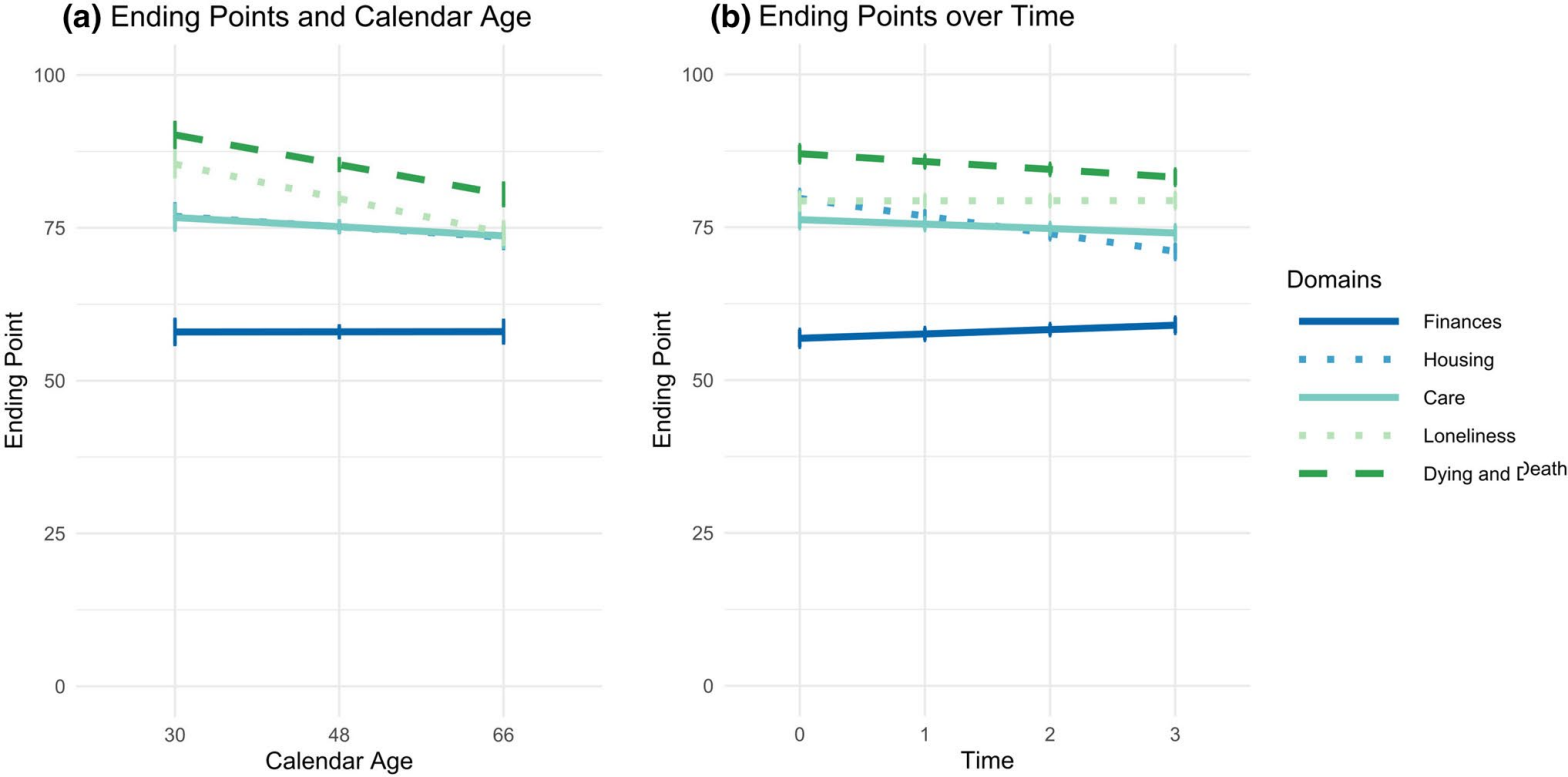
Domain-specific ending points and their relations to time and age. *Notes* Ending points are depicted for the five domains finances, housing, care, loneliness, and dying and death, and can range from 0 (birth) to 100 (death). **a** The domain-specific effects of time on the ending points (Table 2, Model 3), **b** the domain-specific relations between calendar age and the ending points (Table 2, Model 4)

An older age was significantly associated with earlier ending points across domains, *γ*_Age_ = −0.15, SE = 0.05, *p* < .001 (Table 2, Model 2). When relations between calendar age and ending points were investigated specifically for the five domains (Table 2, Model 3), the relation was nonsignificant in the reference domain of finances, *γ*_Age_ = *γ*_Age:Finances_ = 0.00 (SE = 0.05, *p* = .997). The relation to calendar age was however significantly stronger in the four other domains (Fig. 4a; Table 2, Model 3). Particularly, older participants chose earlier ending points for preparing for social connectedness (i.e., preparing against loneliness), and for end of life than younger participants (Fig. 4a). None of the covariates, all *p* values > .050, was significantly related to the ending points.

The time trends in the ending points differed by domain (Table 2, Model 4). Ending points in the reference domain of finances were set increasingly later over time, *γ*_Time_ = *γ*_Time:Finances_ = 0.72, SE = 0.35, *p* = .042. In the domains housing, care, and end of life, the time effects were however significantly more negative, indicating that in these domains ending points were set increasingly earlier over time (Fig. 4b). Again, the most pronounced change appeared in the domain of housing, for which ending points decreased by *γ*_Time:Housing_ = *γ*_Time_ + *δ*_Time:Housing_ = 0.72 + (− 3.60) = −2.88 points per year, and hence by − 8.94 points over the 6 years of the study.

**Fig. 5:**
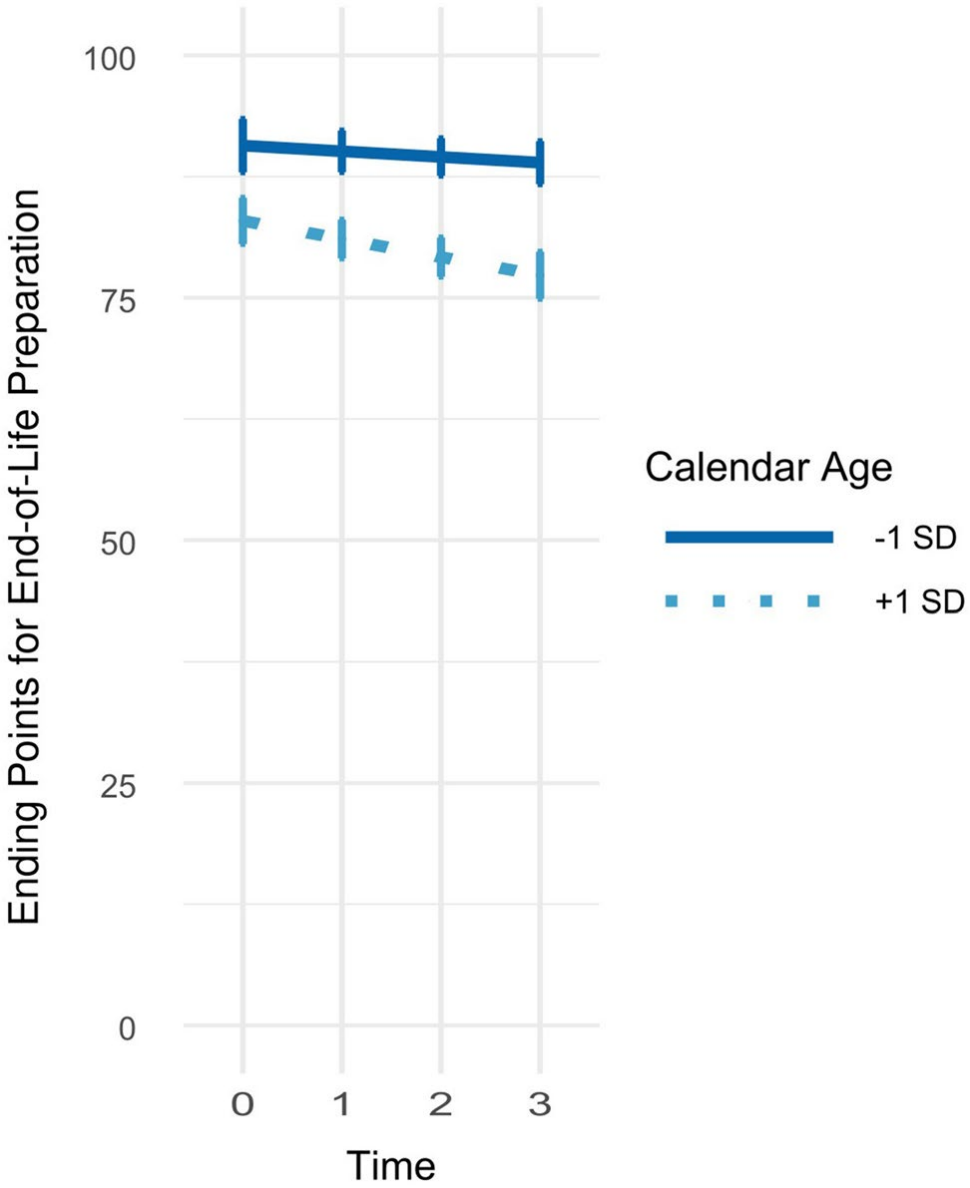
Age-specific time trends in ending points in preparation for dying and death. *Notes* Ending points can range from 0 (birth) to 100 (death). The figure shows how time trends in the ending points for dying and death preparation are influenced by calendar age (Table 2, Model 5)

Calendar age did not affect general time trends in the ending points, *γ*_Time * Age_ = 0.00, SE = 0.01, *p* = .905 (Table 2, Model 4). When investigated specifically for the five domains, only in the end-of-life domain the linear time trend in the ending points interacted with age, *δ*_Time * Age:EndofLife_ = − 0.05, SE = 0.02, *p* = .041 (Table 2, Model 5). Precisely, older participants set increasingly earlier ending points in the domain of end-of-life preparation over time, whereas the ending points set by younger adults remained rather stable (see Fig. 5). Allowing such domain-specific interactions between age and time did however not increase the model fit as indicated by AIC, BIC, and marginal *R*^2^.

Taken together, just as starting points, ending points for aging preparation were set differently depending on the domain and were set the earliest for the domain of finances. Over the course of the study, ending points for aging preparation became earlier in the domains of housing, care, and end of life, but later in the domain of financial planning. An older calendar age was related to overall earlier ending points. Age did not affect general time trends, but seemed to affect time trends specifically in the domain of end of life preparation. Altogether, 29.2% of variance in the starting points could be explained.

When results regarding starting and ending points for late-life preparation are taken together, older adults chose narrower deadlines (i.e., later starting and/or earlier ending points) than younger adults in the domains care, connectedness, and end of life. The deadlines chosen for the domains finances and housing were however comparable across the age range of our sample. Over time, deadlines in most domains were preponed and slightly dilated by participants across age groups. Only in the end-of-life domain, deadlines became earlier for older adults, but later and narrower for younger adults.

## Discussion

Preparing for old age is a matter of optimal timing. Findings of our research suggest that perceived deadlines for aging preparation, defined as the first and the last adequate points to begin with preparation, remained fairly stable across a 6-year time interval. However, there were also changes: deadlines were mostly preponed over four measurement occasions in a 6-year time interval. There were also age-related differences in preparation deadlines from early to late adulthood, but no age by time effects except for end-of-life preparation. In general, older adults as compared to young adults reported more narrow deadlines for the beginning of aging preparation with later starting points and earlier ending points. Besides the relation between a narrower future time perspective and the preference of later starting points, none of the covariates was related to perceived personal deadlines. This suggests that perceived personal deadlines for late life preparation may be shaped by the objective life position, rather than the subjective life position (e.g., subjective age, self-rated health), or structural characteristics (e.g., household net income, marital status). There are three central findings that will be discussed in detail, that is, (a) deadlines differ by calendar age, (b) deadlines of preparation vary by domain, and (c) perceived deadlines are moderately stable over a time interval of 6 years with few exceptions, including age-differential change in end-of-life preparation.

### Deadlines of preparation differ by calendar age

Our findings suggest that calendar age can add to explaining some of the observed variance in perceived personal deadlines for aging preparation. Overall, older adults have more narrow deadlines on when one should have begun with preparing for old age. It cannot be ruled out though that some of such associations with calendar age in fact should be attributed to differences of birth cohorts. For example, many of the older adults may have experienced times of instability in society and economic crisis, whereas young adults may not have experienced upheavals or collapse of societal institutions, and may thus be more likely to think that preparation can be postponed to later years (Settersten 2003). However, the observed changes across the 6 years of measurement suggest that deadlines for preparation become earlier over time in all age groups from early to late adulthood. Therefore, it may be that setting deadlines earlier and narrower is generally associated with getting older. Such findings speak to socioemotional selectivity theory (Carstensen et al. 1999; Lang and Carstensen 2002), which posits that with approaching the ending of life, individuals become more focused on what is considered meaningful and rewarding for the self. The optimal time for late-life preparation may therefore be assigned to midlife rather than to old age as it is something one may not want to spend time with when time is precious and scarce. Rather, one may want to enjoy the positive outcomes of one’s earlier preparation activities, when there is not much time left in one’s life. Consequently, compressed subjective deadlines may be more pronounced when one approaches one’s personal ending of life.

### Deadlines of preparation differ by domain

Personal deadlines of preparation also differed depending on the domain of the preparation activity. For example, findings show that financial preparation for late life was associated with narrower deadlines, whereas preparing for social connectedness and preparing for end of life was related with more dilated deadlines. One implication is that there is a strong consensus between young and old adults as well as over time that financial preparation should be started early in adulthood, whereas there are stronger age and time differences with regard to deadlines for other preparatory domains. Such observations shed light on previous findings on the domain specificity of aging preparation (Kornadt and Rothermund 2014; Kornadt et al. 2018). For example, it was suggested that preparing for a more socially active Third Age differs from preparation for a Fourth Age which may be more strongly associated with functional loss and physical restrictions (Kim-Knauss and Lang 2020). Our analyses of the patterns of personal deadlines point to an additional determinant that may create domain-specific differences in aging preparation: Some investments in preparing for later adulthood can also prove beneficial in earlier phases of adulthood. Saving money, for example, generally increases financial flexibility and security, similarly, the building up of social relationships involves many immediate benefits throughout adulthood, and not only in late life. It may not always be possible for young adults to save money, but it may still be perceived as beneficial to have savings available throughout adulthood. In contrast, other preparatory investments may typically benefit one’s quality of life better when potential age-related functional changes and vulnerability occur and require to adapt one’s living arrangement or caregiving needs. Domain specificity in planning for aging preparation may thus reflect differences in individual perceptions of continuity and discontinuity in the gain–loss dynamics across adulthood. While some domains of preparation activities target on the maintenance or even optimization of functioning in everyday life, for example, with regard to financial planning or social connectivity, other domains of preparation are more strongly associated with coping with discontinuities in everyday functioning, and the adaptation of live circumstances to possible losses, for example, when having to arrange for a possible need of care or new housing.

Our analyses did not allow to explicitly test for the extent to which domain-specific deadlines differed between age groups. However, we observed domain-specific age effects (age * domain; see Table 2; Fig. 4a) suggesting that younger adults compared to older adults might differentiate somewhat more strongly between ending points for the five domains. For example, younger adults perceived wider deadlines for preparation of end-of life, while older adults perceived narrower deadlines in this preparation domain. One possible implication is that in early adulthood, planning of end-of-life issues may be more strongly associated with assumptions of discontinuity and loss. For example, young adults may think that there is still time to prepare for end-of-life issues, when one is old. In contrast, older adults may adopt the view that endings of life can come unexpectedly at any time in life, and that it is therefore better to prepare for one’s end-of-life issues and decisions in midlife rather than in old age.

### Stability and change of perceived deadlines

Generally, perceived personal deadlines did not change much across the four measurement occasions spanning the course of 6 years. The mean-level and rank-order stabilities of the perceived personal deadlines were moderate over the 6 years of the study, and surprisingly consistent across the three 2-year time intervals in our study. This finding points to the general reliability of our assessment of such perceived deadlines, and it also suggests that individuals have a clear and robust understanding of adequate personal deadlines for late-life preparation in different domains. However, there were also a few domain-specific changes in personal deadlines. For example, most people set the perceived deadlines of preparing for housing and of preparing for need of care earlier after 6 years. This finding suggests that there are some domains that over time appear to require a longer preparation in one’s lifetime, and that thus need to be started earlier in life. There were no age * time interaction effects with one notable exception of an age by time effect on the perceived deadlines for end-of-life preparation. Young adults were likely to postpone the starting points for end-of-life preparation over time, while older adults were more likely to set earlier starting points of preparing for end-of-life over the time interval of 6 years. One implication is that such changes accentuate some of the observed cross-sectional age differences suggesting a narrowing of deadlines for end-of-life preparation across adulthood.

### Limitations

Some caveats ought to be considered when interpreting the findings of this research. A first limitation relates to the short 2-year time intervals between the four measurement occasions. Due to the limited time period our study covers and the fact that there were slight changes in the wording of our questionnaires during this time, we cannot fully rule out the possibility that there might be alternative explanations for the linear time trends we discovered. Nevertheless, we observed weak to moderate stability in the response patterns across the measurement occasions, which may reflect stable social norms with regard to some of the reported deadlines, and possibly cohort differences. For example, deadlines of financial preparation were reported as most stable with few or no age differences. Such stability may also reflect the fact that most people have a strong trust in pension insurances and social security systems in Germany. In this regard, cross-cultural comparisons will shed further light on the extent to which the observed stability of perceived deadlines is also reflective of differences in the respective welfare regimes of a specific culture.

Another caveat refers to the fact that there was no information on the extent to which the individuals were compliant with their own perceived deadlines or not. For example, it would be good to know whether individuals actually follow their personal timetables of perceived deadlines and what happens if they do not hold on to them. One implication is that people may seek to achieve a sense of self-consistency by accommodating their personal deadlines over time by subjectively postponing the perceived deadlines in cases when they have not begun with preparation but are after the deadline (Brandtstädter and Rothermund 2003). However, when having started with preparations “in time”, one may realize that having started earlier would have been even better. Thus, information on the status of accomplished preparatory activities may contribute to an improved understanding of the observation that the personal deadlines for preparation were preponed over time and with older age.

Another caveat that needs to be considered when interpreting the findings refers to the linear time effects on the mean levels of preponed deadlines that we observed across 6 years. When extrapolating such linear effects to a larger time frame, the preponement effects on starting points may become unrealistic low. Additional tests of quadratic effects in the data did suggest though that the preponement effects may level out (see footnote 3). We submit that the size and shape of the preponement effects reflect contextual influences that we have not measured in the current study. However, cross-cultural comparisons of these effects promise to shed light on possible macro-structural and culture-associated effects on changes of personal deadlines of late-life preparation over time.

In sum, findings show that perceived personal deadlines for beginning late-life preparation are set narrower and earlier by older as compared to younger adults. Over time the perceived personal deadlines show stability, but also some domain-specific variation and change that suggest that individual are adapting their deadlines of preparation to the challenges and needs of their respective age-related contexts.

## Electronic supplementary material

Below is the link to the electronic supplementary material.

Supplementary material 1 (DOCX 15 kb)

## Footnotes

1 The scope and exact formulation of the questions changed slightly across the four measurement points. Particularly, the second and third measurement point used formulations such as “dealing with the topic of living arrangements in old age” instead of the formulation “preparing for living arrangements in old age”, which was used for the first and fourth measurement point. In order to ensure that this change in wording did not affect the answers of the participants, as well as the results of our analyses, we tested a method factor in the final models. This method factor explained 0.3% of additional variance in the starting point model, and 0.0% of additional variance in the ending point model. Furthermore, the effects of the covariates, main variables, and interactions remained unchanged with the only exception being that the time effect for the starting points in the domain of finances was now reaching significance, *γ*_Time:Finances_ = −2.29 (SE = 0.57, *p *< .001). These results and further descriptive inspections lead us to the assumption that the wording effect was rather marginal and unsystematic.

2 The main effects *γ*_Time_, *γ*_Age_, *γ*_FTP_, and *γ*_Subjective Age_ depict the effect across all domains if no domain interactions are entered. As soon as such domain interactions are entered into the regression models, the main effects refer to the reference domain of finances, e.g., *γ*_Age_ = *γ*_Age:Finances_ in Models 3–5 and *γ*_Time_ = *γ*_Time:Finances_ in Models 4 and 5. The interaction effects of the domains now give deviations from the reference effects in the domain of finances, e.g., *δ*_Age:Housing_, *δ*_Age:Care_, *δ*_Age:Loneliness_, and *δ*_Age:EndofLife_. The total effects within the domains can for example be calculated by *γ*_Age:EndofLife_ = *γ*_Age_* + δ*_Age EndofLife_.

3 Exploratorily, we tested for quadratic time trends in the starting points by using the squared time variable. The quadratic time trend reached significance, *γ*_Time2_ = 1.19, SE = 0.23, *p* < .001, was independent from the wording effect, and indicated a slight flattening of the linear decrease over time.

4 We also tested for quadratic time trends in the ending points by using the squared time variable. The quadratic time trend reached significance, *γ*_Time2_ = −2.55, SE = 0.23, *p* < .001, was independent from the wording effect, and indicated a slightly concave but decreasing time trend.
