# Supplementary material for: Perceived personal deadlines for late-life preparation across adulthood
Source: Eur J Ageing. 2020 Sep 28;18(2):227–38. doi: 10.1007/s10433-020-00581-8 (PMC8217477; doi:10.1007/s10433-020-00581-8)
Supplement: Supplementary file 1 — Supplementary material 1 (DOCX 15 kb) [file 10433_2020_581_MOESM1_ESM.docx]

| **Supplementary Table S1** | | | | | | | | | | | | | | |
| --- | --- | --- | --- | --- | --- | --- | --- | --- | --- | --- | --- | --- | --- | --- |
| *Bivariate Correlations between Starting Points, Ending Points, and Main Predictors and Covariates* | | | | | | | | | | | | | | |
| Measure | 2 | 3 | 4 | 5 | 6 | 7 | 8 | 9 | 10 | 11 | 12 | 13 | 14 | 15 |
| 1. SP Finances | .18** | .30** | .17** | .15** | .33** | -.02 | .05 | -.04 | -.09* | .08* | -.12* | -.02 | -.12* | .05 |
| 2. SP Housing | – | .33** | .17** | .27** | -.05 | .46** | .09* | -.04 | .07 | .14** | -.10* | -.06 | .06 | .05 |
| 3. SP Care |  | – | .26** | .34** | .08 | .17** | .47** | .03 | .06 | .16** | -.13* | -.05 | .04 | .01 |
| 4. SP Loneliness |  |  | – | .24** | .03 | -.04 | .05 | .06 | -.09* | .21** | -.11* | -.07 | -.00 | -.13* |
| 5. SP Dying |  |  |  | – | -.07 | -.01 | -.01 | -.15** | .16** | .32** | -.11* | -.18** | .14* | .01 |
| 6. EP Finances |  |  |  |  | – | .30** | .41** | .33** | .25** | -.09* | -.05 | .03 | -.14* | .05 |
| 7. EP Housing |  |  |  |  |  | – | .55** | .39** | .40** | -.16** | .07 | .05 | -.03 | .12* |
| 8. EP Care |  |  |  |  |  |  | – | .51** | .45** | -.19** | .05 | .09* | -.03 | .12* |
| 9. EP Loneliness |  |  |  |  |  |  |  | – | .47** | -.33** | .14* | .19** | -.04 | .05 |
| 10. EP Dying |  |  |  |  |  |  |  |  | – | -.25** | .08 | .11* | -.05 | .12* |
| 11. Calendar Age |  |  |  |  |  |  |  |  |  | – | -.46** | -.33** | .21** | -.22** |
| 12. FTP |  |  |  |  |  |  |  |  |  |  | – | -.12* | .27** | .40** |
| 13. Subjective Age |  |  |  |  |  |  |  |  |  |  |  | – | -.26** | -.21** |
| 14. PLE |  |  |  |  |  |  |  |  |  |  |  |  | – | .29** |
| 15. Self-Rated Health |  |  |  |  |  |  |  |  |  |  |  |  |  | – |
| *Notes.* SP = starting point; EP = ending point; FTP= future time perspective; PLE = perceived life expectancy.  **p* < .05. ***p* < .001. | | | | | | | | | | | | | | |
